# Diagnostic Value of Serum VEGF-D in Lymphangioleiomyomatosis: Results of the First Prospective Study in the Russian Federation

**DOI:** 10.3390/diagnostics16040533

**Published:** 2026-02-11

**Authors:** Marina Makarova, Gulsara Baimakanova, Andrey Belevskiy, Anzhelika Chegodar, Airat Bilyalov, Natalia Bodunova

**Affiliations:** 1 Federal State Autonomous Educational Institution of Higher Education “N.I. Pirogov Russian National Research Medical University”, Ministry of Health of the Russian Federation, 117513 Moscow, Russia; 2 Pulmonology Scientific Research Institute, Federal Medical and Biological Agency of the Russian Federation, 115682 Moscow, Russia; 3 City Clinical Hospital Named After S.S. Yudin, Moscow Department of Health, 115487 Moscow, Russia; 4 Loginov Moscow Clinical Scientific Center, 111123 Moscow, Russia; 5 Life Improvement by Future Technologies (LIFT) Center, 121205 Moscow, Russia

**Keywords:** lymphangioleiomyomatosis, VEGF-D, serum biomarker, cystic lung disease, rare lung disease, noninvasive diagnosis

## Abstract

**Background/Objectives**: Lymphangioleiomyomatosis (LAM) is a rare cystic lung disease for which serum vascular endothelial growth factor D (VEGF-D) is a recommended diagnostic biomarker. Data from the Russian Federation remain limited. We aimed to evaluate the diagnostic accuracy of VEGF-D in women with multiple pulmonary cysts and to assess diagnostic thresholds in a Russian cohort. **Methods**: In a single-center prospective cohort study, 71 women aged 20–74 years with multiple lung cysts on high-resolution computed tomography were enrolled. Serum VEGF-D levels were measured using Quantikine ELISA. Diagnoses were established according to international guidelines. Receiver operating characteristic analysis was used to evaluate diagnostic performance and identify cut-off values. **Results**: Of 71 patients, 48 (68%) had definite LAM. VEGF-D levels were higher in LAM than in non-LAM patients (1425 ± 872.1 pg/mL vs. 552 ± 276.5 pg/mL, *p* < 0.0001). The area under the curve was 0.866 (95% CI 0.783–0.950). A cohort-derived threshold of 738 pg/mL yielded 81% sensitivity and 79% specificity. VEGF-D levels of 800 pg/mL or higher were observed in 75% of LAM cases. **Conclusions**: Serum VEGF-D demonstrates high diagnostic value for LAM in women with multiple pulmonary cysts in the Russian Federation. The findings support implementation of VEGF-D testing in routine practice and are consistent with use of the internationally recommended 800 pg/mL threshold for noninvasive confirmation in most patients.

## 1. Introduction

Lymphangioleiomyomatosis (LAM) is currently regarded as a low-grade metastatic neoplasm characterized by the proliferation of abnormal smooth muscle-like cells known as LAM cells [1].

LAM can arise spontaneously as a result of somatic mutations, most often in the TSC2 (tuberous sclerosis complex) gene (sporadic LAM) or as part of the broader phenotype of the genetic disorder tuberous sclerosis (TSC-associated LAM) [2,3].

Sporadic LAM and TSC are rare diseases. The true prevalence of LAM is difficult to determine. According to Harknett E. C. and colleagues, the prevalence of LAM ranges from 3 to 8 cases per 1 million women [4], while a study by Evelyn Lynn and colleagues reported at least 21 cases per 1 million women worldwide [5]. Several studies have noted the prevalence of LAM among women with TSC (mainly with TSC2 mutations), which varies from 26 to 50 percent [6].

### 1.1. Pathogenesis

The pathogenesis of LAM is that deletion of the tuberous sclerosis complex (TSC) genes leads to overactivation of the mammalian target of rapamycin pathway (mTOR) [7], which promotes the proliferation of LAM cells [8].

LAM cells have an unknown origin, and they have been found in the bloodstream, urine, and pleural and abdominal effusions of affected patients. Through the lymphatic system or bloodstream, LAM cells infiltrate the lungs, causing characteristic cystic lesions, or the abdomen, causing tumors called angiomyolipomas (AMLs), generally involving the kidneys or the lymphatic vessels, giving origin to lymphangioleiomyomas [9,10].

### 1.2. Serum VEGF-D as a Biomarker

Vasculogenesis is an important stage in physiological and pathological processes in the body. At present, vascular endothelial growth factors (VEGF) are considered key regulators of vessel formation. Mammals express VEGF-A, VEGF-B, VEGF-C, VEGF-D and placental growth factor (PlGF). These factors can bind to three types of VEGF receptors (VEGFR 1, VEGFR 2, VEGFR 3) with distinct affinities and specificities, which leads to different biological responses. VEGFR 1 and VEGFR 2 are found on vascular endothelial cells and, in some cases, on non-endothelial cells, while VEGFR 3 is predominantly expressed on lymphatic endothelial cells [11].

VEGF-D is a secreted glycoprotein that is expressed in the lungs, heart, skeletal muscles and intestines of healthy individuals [12]. Fundamental research demonstrates its potential role in oncologic, cardiovascular and ophthalmologic diseases as a factor that promotes angiogenesis, which is the formation and growth of new blood vessels, and lymphangiogenesis, which is the growth of lymphatic vessels, through activation of VEGFR 2 and VEGFR 3 [13]. This article focuses on VEGF-D because LAM cells specifically produce this molecule [14].

### 1.3. Studies on LAM and the Diagnostic Utility of VEGF-D

Until 2006, serum VEGF-D levels had not been examined in LAM. Early research focused on different VEGF classes. Seyama K. and colleagues used an ELISA assay to measure VEGF-A, VEGF-C, and VEGF-D concentrations in the serum of 44 patients with LAM. Only VEGF-D levels were significantly elevated compared with age- and sex-matched healthy controls [14]. Young L. R. and colleagues were the first to demonstrate the diagnostic value of serum VEGF-D in LAM compared with other diffuse cystic lung diseases [15,16].

A large international multicenter trial on the efficacy of sirolimus in lymphangioleiomyomatosis (MILES) evaluated serum VEGF-D as a biomarker of disease severity and treatment response [17]. The diagnostic value of VEGF-D was later confirmed in several additional LAM cohorts [18,19,20].

In 2016, clinical guidelines from the American Thoracic Society and the Japanese Respiratory Society recommended serum VEGF-D testing as a diagnostic tool for all patients with typical LAM-like pulmonary cysts on CT before considering surgical lung biopsy when no other confirmatory features of LAM are present. Confirmatory features include tuberous sclerosis, angiomyolipomas, lymphangioleiomyomas, and chylous pleural effusion or ascites [21,22].

When lymphangioleiomyomatosis is suspected on clinical and radiological grounds, morphological verification is not required in all situations. Current diagnostic approaches emphasize the use of the least invasive methods. According to clinical guidelines for the diagnosis and treatment of lymphangioleiomyomatosis, a definitive diagnosis can only be established through histological examination of lung tissue if there is no history of tuberous sclerosis, chylous pleural effusion or ascites, lymphangioleiomyomas or angiomyolipomas [23]. VEGF-D is recognized worldwide as a laboratory biomarker of lymphangioleiomyomatosis with a diagnostic threshold of 800 pg per milliliter or higher [1]. This test was not available in the Russian Federation until April 2023. In this study we evaluated the diagnostic value of serum VEGF-D in patients with lymphangioleiomyomatosis living in the Russian Federation.

## 2. Materials and Methods

### 2.1. Study Design and Participants

A single-center prospective cohort study enrolled 71 women aged 20 to 74 years who had multiple air-filled pulmonary cystic lesions on high-resolution computed tomography of the chest. Eligibility required an age of 18 years or older and evidence of multiple lung cysts on HRCT. Exclusion criteria were age under 18 years and absence of cystic lung involvement on HRCT. All participants gave written informed consent. The study protocol was approved by the local ethics committee of the A.S. Loginov Moscow Clinical Research Center. The approval number of the committee is 46426/16.7. The patient recruitment period was from 4 January 2024 to 19 December 2024.

### 2.2. Case Definition and Clinical Data Collection

A definite diagnosis of lymphangioleiomyomatosis was assigned when a typical pattern of pulmonary cysts on HRCT was accompanied by histological confirmation or at least one supportive extrapulmonary feature according to the European Respiratory Society diagnostic and management criteria. Supportive features included tuberous sclerosis complex, renal angiomyolipoma, lymphangioleiomyoma, and chylous pleural effusion or ascites. Clinical data were collected using a standardized case report form by trained investigators. Demographic variables, smoking history, respiratory symptoms, prior treatments including mTOR inhibitors, history of extrapulmonary manifestations, and results of prior investigations were recorded.

### 2.3. Radiological Assessment

HRCT examinations were performed on a multi-detector CT scanner using thin collimation. Scans were reconstructed with a 1 mm slice thickness in a lung window. Two thoracic radiologists with over five years of experience reviewed all scans independently. In the event of disagreement, a consensus reading was obtained.

### 2.4. Biospecimen Collection and Preanalytical Handling

Peripheral venous blood was collected from fasting participants in the morning into serum separator tubes. Samples were allowed to clot at room temperature for 30 min and then centrifuged at 2000× *g* for 10 min. Serum was aliquoted into 0.5 to 1 mL portions and stored at minus 80 degrees Celsius until assay. Time from blood draw to freezing was recorded for each sample. Samples that underwent more than one freeze–thaw cycle were excluded from the primary analysis.

### 2.5. VEGF-D Measurement

Serum VEGF-D concentration was measured using the Human VEGF-D Quantikine ELISA kit from R and D Systems, USA, according to the manufacturer’s instructions. Key assay parameters are reported here to ensure reproducibility. All samples were assayed in duplicate. Samples with optical density values outside the range of the standard curve were re-assayed after appropriate dilution. Each plate included a seven-point calibration curve prepared from the recombinant human VEGF-D standard supplied with the kit and two quality control sera at low and high concentrations. The assay’s lower limit of detection is reported by the manufacturer as 31.3 pg per milliliter. Inter-assay and intra-assay coefficients of variation were monitored using quality control samples and were maintained below 15 percent and 10 percent, respectively. Assays were performed by laboratory staff who were blinded to the clinical diagnosis.

### 2.6. Data Management and Statistical Analysis

Clinical and laboratory data were entered into a secure electronic database with predefined range checks. Statistical analyses were performed using SPSS for Windows version 18 and R version 4.1 for additional procedures. Continuous variables are presented as mean plus or minus standard deviation or as median with interquartile range when distributions were skewed. Categorical variables are presented as counts and percentages. Normality of continuous variables was assessed with the Shapiro–Wilk test. Between-group comparisons for continuous variables were performed with the Mann–Whitney U test for non-normal distributions and with Student’s *t*-test when normality assumptions were met. Categorical variables were compared with the chi-square test or Fisher’s exact test, as appropriate.

### 2.7. Diagnostic Performance and Threshold Selection

Diagnostic performance of serum VEGF-D for distinguishing definite lymphangioleiomyomatosis from other causes of multiple pulmonary cysts was evaluated by receiver operating characteristic analysis. The area under the ROC curve with a 95 percent confidence interval was calculated using the DeLong method. Sensitivity and specificity were calculated for prespecified thresholds of 738 pg per milliliter and 800 pg per milliliter. The optimal threshold in our cohort was also determined by maximizing the Youden index. The positive predictive value and negative predictive value were reported for observed disease prevalence in the study sample.

## 3. Results

The study included 71 women with a mean age of 48.7 ± 12.3 years and a mean body mass index of 25.17 ± 5.6 kg/m^2^. Among them, 48 women, which is 68 percent, were diagnosed with lymphangioleiomyomatosis with a mean age of 48.2 ± 12.9 years and a mean body mass index of 24.8 ± 5.8 kg/m^2^. All patients with lymphangioleiomyomatosis had a definite diagnosis established in accordance with current diagnostic and treatment guidelines [1]. Sporadic lymphangioleiomyomatosis was present in most cases, which was 47 patients, or 98 percent, while only one patient, or 2 percent, had tuberous sclerosis-associated disease.

**Figure 1:**
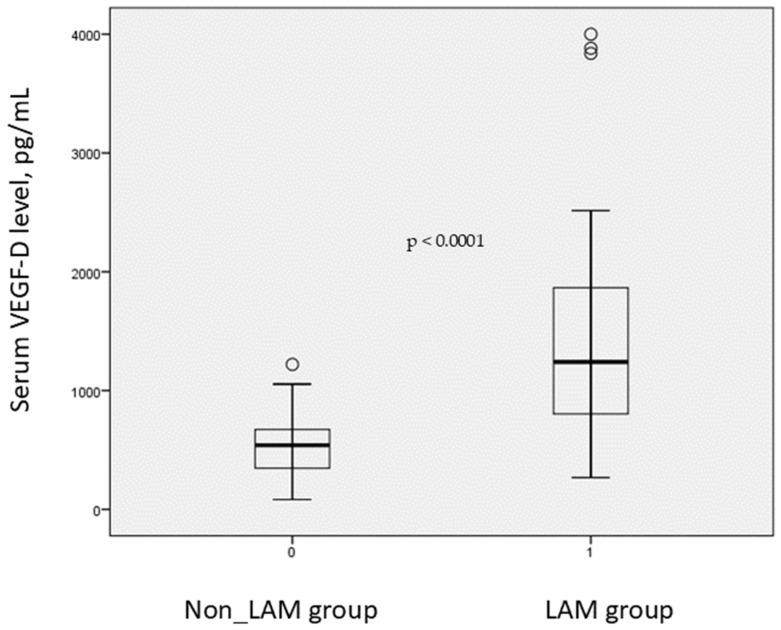
Serum VEGF-D in patients with and without LAM. Box plots indicate the median and interquartile range, whiskers represent the most extreme values within 1.5 interquartile ranges, and circles denote outliers. The *p*-value for the between-group comparison is shown in the figure (*p* < 0.0001).

The group of women with multiple pulmonary cysts not associated with LAM, which included 23 patients (32%), was comparable to the LAM group in terms of age and body mass index. The mean age was 49.8 ± 12.9 years, with *p* > 0.576, and the mean body mass index was 26.2 ± 4.9 kg/m^2^, with *p* > 0.309. Serum VEGF-D levels were significantly higher in patients with LAM compared with those without the disease, with values of 1425 ± 872.1 pg/mL versus 552 ± 276.5 pg/mL with *p* < 0.0001 (Figure 1). Patients with LAM and extrapulmonary manifestations, which included 23 women, showed a trend toward higher serum VEGF-D concentrations compared with those with isolated pulmonary involvement, which included 25 women with values of 1510 ± 968.6 pg/mL versus 1328.5 ± 761.0 pg/mL, with *p* < 0.06. An elevation of VEGF-D at or above 800 pg/mL was identified in 75 percent of patients with LAM, which corresponded to 36 cases.

**Figure 2:**
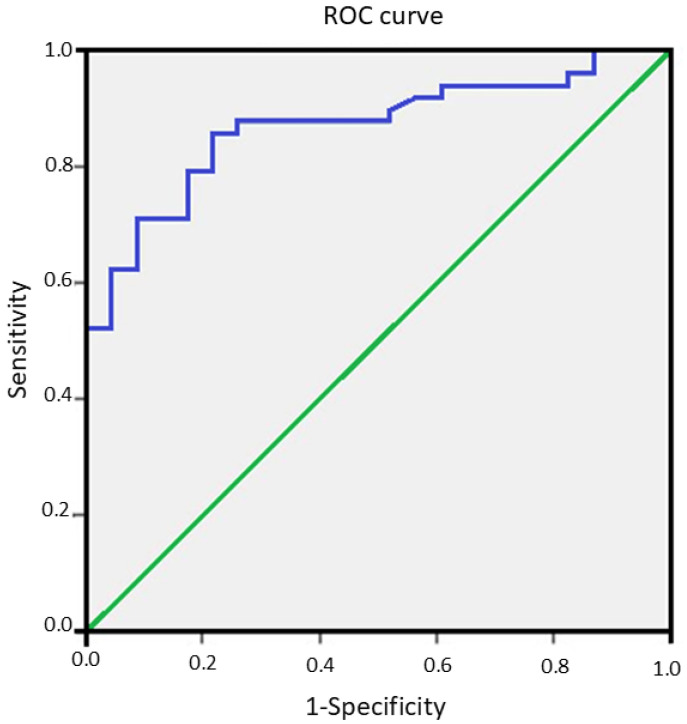
Receiver operating characteristic curve for serum VEGF-D used to distinguish LAM from other causes of multiple pulmonary cysts. The curve demonstrates high diagnostic accuracy, with an area under the curve of 0.866. Green diagonal line shows the no-discrimination reference, equivalent to a classifier performing at random. The blue line is the ROC curve, and the AUC is the area under this curve relative to the diagonal reference line.

To assess the diagnostic value of this marker in our cohort, a receiver operating characteristic curve was constructed, as shown in Figure 2. The area under the curve for VEGF-D in LAM was 0.866 with a 95 percent confidence interval of 0.783 to 0.950, with *p* < 0.0001. A threshold value of 738 pg/mL demonstrated a sensitivity of 81 percent and a specificity of 79 percent for the diagnosis of definite LAM.

In our ROC analysis, the cohort-derived VEGF-D threshold for LAM diagnosis was 738 pg/mL, providing a sensitivity of 81%. Importantly, three patients with definite LAM had VEGF-D concentrations below the guideline-recommended diagnostic threshold of 800 pg/mL, specifically, 789, 751, and 796 pg/mL. All three had a typical HRCT pattern consistent with LAM; in two patients, the diagnosis was confirmed by surgical lung biopsy, and in one patient, renal angiomyolipoma was documented. These cases highlight that, while VEGF-D demonstrates good overall diagnostic performance in our cohort, values below 800 pg/mL do not exclude LAM in the presence of a typical HRCT pattern and or other confirmatory features, and results should be interpreted in their full clinical context.

Full information on the characteristics of the patients is provided in Table 1.

The detailed diagnoses and diagnostic status of patients included in the non-LAM group are provided in Supplementary Table S1.

## 4. Discussion

In our study, serum VEGF-D levels were measured for the first time in the Russian Federation in patients who had multiple air-filled pulmonary cysts identified on high-resolution computed tomography of the chest. The first recommendations for the evaluation of this laboratory marker before diagnostic lung biopsy in patients with suspected LAM with diffuse pulmonary cysts and without extrapulmonary manifestations were published in 2016 in the official clinical guidelines of the American Thoracic Society and the Japanese Respiratory Society. These guidelines reference seven studies that were conducted mainly in the United States and Europe [1]. More recent evidence comes from a meta-analysis that included a larger number of studies with broader geographical coverage. Li et al. in 2022 [24] reported a pooled sensitivity of 0.82 and a specificity of 0.98 with an area under the summary receiver operating characteristic curve of approximately 0.98, which confirms the high diagnostic accuracy of the marker across different cohorts. These values are consistent with the area under the curve of 0.866 observed in our study [24]. The initial prospective study by Young et al. demonstrated that VEGF-D effectively distinguishes LAM from other diffuse cystic lung diseases with high diagnostic performance [16]. Data from the Japanese cohort published by Hirose et al. confirmed high specificity at the 800 pg/mL threshold and good sensitivity at lower thresholds, which supports the reproducibility of the assay across different populations [25].

When LAM is suspected based on clinical and radiological features, a VEGF-D level of 800 pg/mL or higher supports the diagnosis [1]. However, studies included in the meta-analysis reported VEGF-D concentrations ranging from 440 to 1239 pg/mL [24]. This variability may be explained by several factors such as the presence of tuberous sclerosis, lymphatic complications, disease severity, treatment with sirolimus and differences in sample handling, pre-analytical preparation and other technical aspects of the assay [24].

Recent clinical reviews underline that VEGF-D is now integrated into the standard diagnostic algorithm for diffuse cystic lung diseases as the best validated blood biomarker, but also highlight its limited sensitivity and the persistence of a substantial proportion of patients with LAM who have VEGF-D levels below the recommended diagnostic threshold [26]. In a Brazilian reference center cohort, Amaral and colleagues showed that serum VEGF-D concentrations were only weakly correlated with lung function impairment but strongly associated with the extent of lymphatic involvement, and emphasized that local population characteristics and disease phenotype should be considered when interpreting VEGF-D levels [27]. These findings are consistent with our observation that higher VEGF-D levels were predominantly seen in women with chylous complications or renal angiomyolipomas and support the concept that VEGF-D primarily reflects overall lymphatic tumor burden rather than isolated pulmonary dysfunction.

### 4.1. Diagnostic Utility

Our results show that VEGF-D levels of 800 pg/mL or higher were observed in most women with LAM, which accounted for 75 percent. However, 25% of patients had VEGF-D levels below this diagnostic threshold and required surgical lung biopsy for definitive diagnosis. Therefore, a negative result does not exclude LAM, and further diagnostic evaluation is necessary. In our study the threshold value derived from the receiver operating characteristic curve was 738 pg/mL, which predicted the disease in most patients with a sensitivity of 81%.

This cohort-derived cut-off reflects ROC optimization in our single-center dataset and should not be interpreted as a replacement for the guideline-recommended 800 pg/mL threshold used to prioritize specificity. Lowering the threshold may increase sensitivity but is expected to reduce specificity and increase false-positive results in heterogeneous non-LAM populations, potentially leading to unnecessary additional investigations or invasive procedures.

Our findings also show that patients with chylous abnormalities or LAM or renal angiomyolipomas had higher VEGF-D levels compared with those who had isolated cystic lung disease. This pattern reflects greater disease burden and higher secretory activity of LAM cells in patients with extrapulmonary manifestations.

Among patients without confirmed LAM, the verified diagnoses included pulmonary emphysema, sarcoidosis and endometriosis. In most remaining cases, the etiology of lung cysts remained unclear, and additional diagnostic investigations are required.

In patients with multiple pulmonary cysts and clinical suspicion of LAM, particularly when typical HRCT findings are present but other confirmatory features are absent, including tuberous sclerosis complex, chylous pleural effusion or ascites, lymphangioleiomyoma, or renal angiomyolipoma, we recommend serum VEGF-D testing prior to considering lung biopsy. In contrast, when cystic lung disease is the only manifestation and HRCT findings are atypical for LAM, morphological verification of lung tissue should be considered to establish an alternative diagnosis and reduce the risk of misclassification.

Longitudinal studies of sirolimus therapy provide additional context for interpreting VEGF D as a dynamic biomarker. Taveira DaSilva et al. demonstrated that prolonged sirolimus treatment leads to a sustained reduction in serum VEGF-D from markedly elevated baseline levels together with stabilization of FEV1 and diffusing capacity, although the magnitude of VEGF D decline did not directly correlate with changes in lung function [28]. In a larger multicenter cohort, Hu et al. confirmed that sirolimus maintains VEGF-D at lower levels for up to four years while improving or stabilizing pulmonary function, exercise capacity and quality of life, again supporting the role of VEGF-D as a marker of biological target engagement rather than a precise surrogate of functional response [29]. Beyond VEGF-D, recent work has focused on multimarker strategies; Revilla López and colleagues evaluated a panel of analytes related to extracellular matrix remodeling, lymphangiogenesis and angiogenesis and proposed biomarker combinations that may improve diagnostic accuracy, whereas Terraneo et al. reported that matrix metalloproteinases 2 and 7 show promising discrimination between LAM, tuberous sclerosis complex and healthy controls and could complement VEGF-D in future diagnostic algorithms [30,31].

### 4.2. Study Limitations

This study has several limitations, which include the relatively small sample size and the absence of histological verification in a subset of patients. Further research is needed, along with an active strategy aimed at histological confirmation of cystic lung disease. Such efforts will improve diagnostic accuracy and help clarify the role and clinical utility of VEGF-D. To address these limitations, future work should include a multicenter study with broader geographic representation, standardized pre-analytical protocols and validation of the assay in an independent cohort. An additional research direction is the evaluation of VEGF-D dynamics during treatment with mTOR inhibitors and the assessment of its prognostic value as a marker of disease severity. As a single-center prospective study, our cohort may reflect regional referral patterns, including enrichment for patients with more complex diagnostic trajectories and a higher pre-test probability of LAM compared with unselected populations presenting with incidental cystic lung changes. In addition, local differences in case mix, availability and timing of VEGF-D testing, and the prevalence of phenotypic subgroups, such as sporadic LAM versus TSC-associated LAM, may influence diagnostic performance estimates and limit direct extrapolation to other settings and larger international cohorts.

## 5. Conclusions

The present study is the first in the Russian Federation to evaluate the diagnostic significance of serum VEGF-D in women with multiple air-filled pulmonary cysts. The findings confirm the applicability of this biomarker for noninvasive confirmation of LAM and reproduce trends described in international studies. The use of VEGF-D as a laboratory criterion in patients with a typical computed tomography pattern of disease reduces the need for invasive procedures and accelerates diagnostic decision making. These results provide a foundation for integrating VEGF-D testing into clinical practice and for further standardization of the method at the national level.

## Figures and Tables

**Table 1:** Characteristics of the two patient groups.

	LAM Group n = 48	Non-LAM Group n = 23
Age, years mean ± SD	48.2 ± 12.9	49.8 ±11.2
BMI, kg/m^2^ mean ± SD	24.8 ± 5.8	26.2 ± 4.9
Smokers n (%)	18 (38)	5 (22)
Dyspnea n (%)	25 (52)	5 (22)
Chylothorax n (%)	4 (8)	0
Pneumothorax n (%)	15 (31)	3 (13)
Hemoptysis n (%)	3 (6)	0
Extrapulmonary manifestations (renal angiomyolipoma, lymphangioleiomyoma, lymphadenopathy) n (%)	24 (50)	3 (13)
VEGF-D * mean ± SD	1425.0 ± 872.1	550.2 ± 276.5
FEV_1_ *, % pred. mean ± SD	77.2 ± 23.3	98.4 ± 13.9
FVC *, % pred. mean ± SD	90.3 ± 17.6	104.2 ± 13.7
FEV_1_/FVC, % mean ± SD	69.1 ± 16.1	77.7 ± 5.8
DLco *, mL/min/mmHg mean ± SD	66.3 ± 24.5	88.3 ± 14.9

BMI, body mass index; VEGF-D, vascular endothelial growth factor D; FEV_1_, forced expiratory volume in 1 s; FVC, forced vital capacity; DLco, diffusing capacity of the lungs for carbon monoxide. * *p* < 0.05. Three patients in the non-LAM group had renal angiomyolipoma. The final diagnoses were Birt–Hogg–Dubé syndrome (pathogenic FLCN mutation) in one patient, morphologically verified thoracic endometriosis in one patient, and morphologically verified pulmonary emphysema in one patient.

## Data Availability

The data are not publicly available due to restrictions, as they contain information that could compromise the privacy of research participants. Requests to access the additional data should be addressed to the following email: gulsara.bai@mail.ru.

## Supplementary Materials

The following supporting information can be downloaded at: https://www.mdpi.com/article/10.3390/diagnostics16040533/s1, Table S1: The detailed diagnoses and diagnostic status of patients
