# Supplementary material for: Diagnostic Value of Serum VEGF-D in Lymphangioleiomyomatosis: Results of the First Prospective Study in the Russian Federation
Source: Diagnostics (Basel). 2026 Feb 11;16(4):533. doi: 10.3390/diagnostics16040533 (PMC12939067; doi:10.3390/diagnostics16040533)
Supplement: Supplementary file 1 [file diagnostics-16-00533-s001.zip › diagnostics-4038489-supplementary.pdf]

n	Diagnosis	Diagnostic confirmation
1	Pulmonary emphysema	Morphological verification
1	Sarcoidosis	Morphological verification
1	Thoracic endometriosis	Morphological verification
3	Birt–Hogg–Dubé syndrome	Pathogenic mutations identified in the *FLCN* gene
1	Lymphocytic interstitial pneumonia associated with Sjögren's syndrome	Clinical and radiological criteria
4	Undergoing diagnostic workup	Non-informative fiberoptic bronchoscopy with transbronchial cryobiopsy; referred for surgical lung biopsy via video-assisted thoracoscopic surgery
6	Declined morphological verification	Patient refusal
6	Morphological verification not clinically justified	Minimal structural lung involvement without functional impairment
